# Screening for Tay‐Sachs disease carriers by full‐exon sequencing with novel variant interpretation outperforms enzyme testing in a pan‐ethnic cohort

**DOI:** 10.1002/mgg3.836

**Published:** 2019-07-10

**Authors:** Alana C. Cecchi, Elizabeth S. Vengoechea, Kristjan E. Kaseniit, Melanie W. Hardy, Laura A. Kiger, Nikita Mehta, Imran S. Haque, Krista Moyer, Patricia Z. Page, Dale Muzzey, Karen A. Grinzaid

**Affiliations:** ^1^ Myriad Women’s Health South San Francisco California; ^2^ Department of Human Genetics Emory University School of Medicine Atlanta Georgia; ^3^ Present address: Department of Medical Genetics The University of Texas Health Science Center at Houston Houston Texas; ^4^ Present address: Department of Laboratory Medicine and Pathology Mayo Clinic Rochester Minnesota; ^5^ Present address: Department of Clinical and Diagnostic Sciences University of Alabama at Birmingham Birmingham Alabama

**Keywords:** carrier screening, HEXA enzyme testing, Tay‐Sachs disease, variant interpretation, VUS reclassification

## Abstract

**Background:**

Pathogenic variants in *HEXA* that impair β‐hexosaminidase A (Hex A) enzyme activity cause Tay‐Sachs Disease (TSD), a severe autosomal‐recessive neurodegenerative disorder. Hex A enzyme analysis demonstrates near‐zero activity in patients affected with TSD and can also identify carriers, whose single functional copy of *HEXA* results in reduced enzyme activity relative to noncarriers. Although enzyme testing has been optimized and widely used for carrier screening in Ashkenazi Jewish (AJ) individuals, it has unproven sensitivity and specificity in a pan‐ethnic population. The ability to detect *HEXA* variants via DNA analysis has evolved from limited targeting of a few ethnicity‐specific variants to next‐generation sequencing (NGS) of the entire coding region coupled with interpretation of any discovered novel variants.

**Methods:**

We combined results of enzyme testing, retrospective computational analysis, and variant reclassification to estimate the respective clinical performance of TSD screening via enzyme analysis and NGS. We maximized NGS accuracy by reclassifying variants of uncertain significance and compared to the maximum performance of enzyme analysis estimated by calculating ethnicity‐specific frequencies of variants known to yield false‐positive or false‐negative enzyme results (e.g., pseudodeficiency and B1 alleles).

**Results:**

In both AJ and non‐AJ populations, the estimated clinical sensitivity, specificity, and positive predictive value were higher by NGS than by enzyme testing. The differences were significant for all comparisons except for AJ clinical sensitivity, where NGS exceeded enzyme testing, but not significantly.

**Conclusions:**

Our results suggest that performance of an NGS‐based TSD carrier screen that interrogates the entire coding region and employs novel variant interpretation exceeds that of Hex A enzyme testing, warranting a reconsideration of existing guidelines.

## 1 INTRODUCTION

Tay‐Sachs disease (TSD [OMIM #272800]) is an autosomal‐recessive lysosomal storage disorder caused by deficiency of the α‐subunit of the β‐hexosaminidase enzyme (Hex A) resulting in GM2 ganglioside neuronal accumulation. It is characterized by progressive neurodegeneration, leading to early childhood death in individuals with the infantile form and a delayed clinical course in individuals with juvenile‐ or adult‐onset disease. Population‐based carrier screening for TSD began in the 1970s for individuals of Ashkenazi Jewish (AJ) descent, as the frequency of carriers in this group (1 in 33) was found to be elevated compared to the general population (1 in 260) (Gross, Pletcher, Monaghan, & Professional Practice and Guidelines Committee, 2008; Kaback et al., 1993). These early screening programs utilized biochemical analysis of Hex A to identify carriers and successfully reduced the prevalence of TSD by more than 90% in the United States and Canada (Kaback & Zeiger, 1972).

Aside from Hex A enzyme analysis, direct DNA analysis of *HEXA* (OMIM *606869) can also determine TSD carrier status. Targeted testing of three common *HEXA* variants was first established as a useful supplement to enzyme testing in the AJ population, yielding high sensitivity and positive predictive value (PPV) for individuals with AJ ancestry (Triggs‐Raine et al., 1990). While targeted variant panels have since grown to include additional pathogenic variants, the sensitivity of targeted testing is limited by the variants selected—typically those most prevalent in the AJ population—yielding carrier detection rates ranging from 89% to 99% in AJs depending on the population homogeneity and set of variants tested (Bach, Tomczak, Risch, & Ekstein, 2001; Kaback et al., 1993; Schneider et al., 2009; Yoo, Astrin, & Desnick, 1993). Previous studies have shown the sensitivity of targeted variant analysis for common AJ variants to be markedly reduced in pan‐ethnic populations (Kaback et al., 1993; Park et al., 2010). More recently, next‐generation sequencing (NGS) of *HEXA* has been employed as an alternative method for TSD carrier screening because it can identify common, rare, and novel variants (Hoffman et al., 2013). To be classified as pathogenic, a novel variant must undergo a variant‐interpretation process; specifically, it must meet a set of criteria established by the American College of Medical Genetics and Genomics‐Association for Molecular Pathology (ACMG‐AMP) (Richards et al., 2015).

A screening method for TSD that maximizes carrier detection across all ethnicities is necessary and important because the genetic diversity of the U.S. population has been steadily increasing over the last several decades due to increased interethnic marriage rates. Furthermore, the majority of TSD‐affected births is now from couples where at least one partner is non‐AJ (Kaback et al., 1993; Lew, Burnett, Proos, & Delatycki, 2015). Enzyme‐based testing has notable shortcomings in clinical performance for non‐AJ populations, including the lack of well‐established detection rates and reference ranges (Mehta et al., 2016). Nevertheless, the American College of Obstetricians and Gynecologists (ACOG) states that enzyme testing may be preferable for patients from low‐risk populations (Committee on Genetics, 2017), and ACMG endorses enzyme testing as the more reliable carrier screening method across all ethnicities (Edwards et al., 2015).

**Figure 1:**
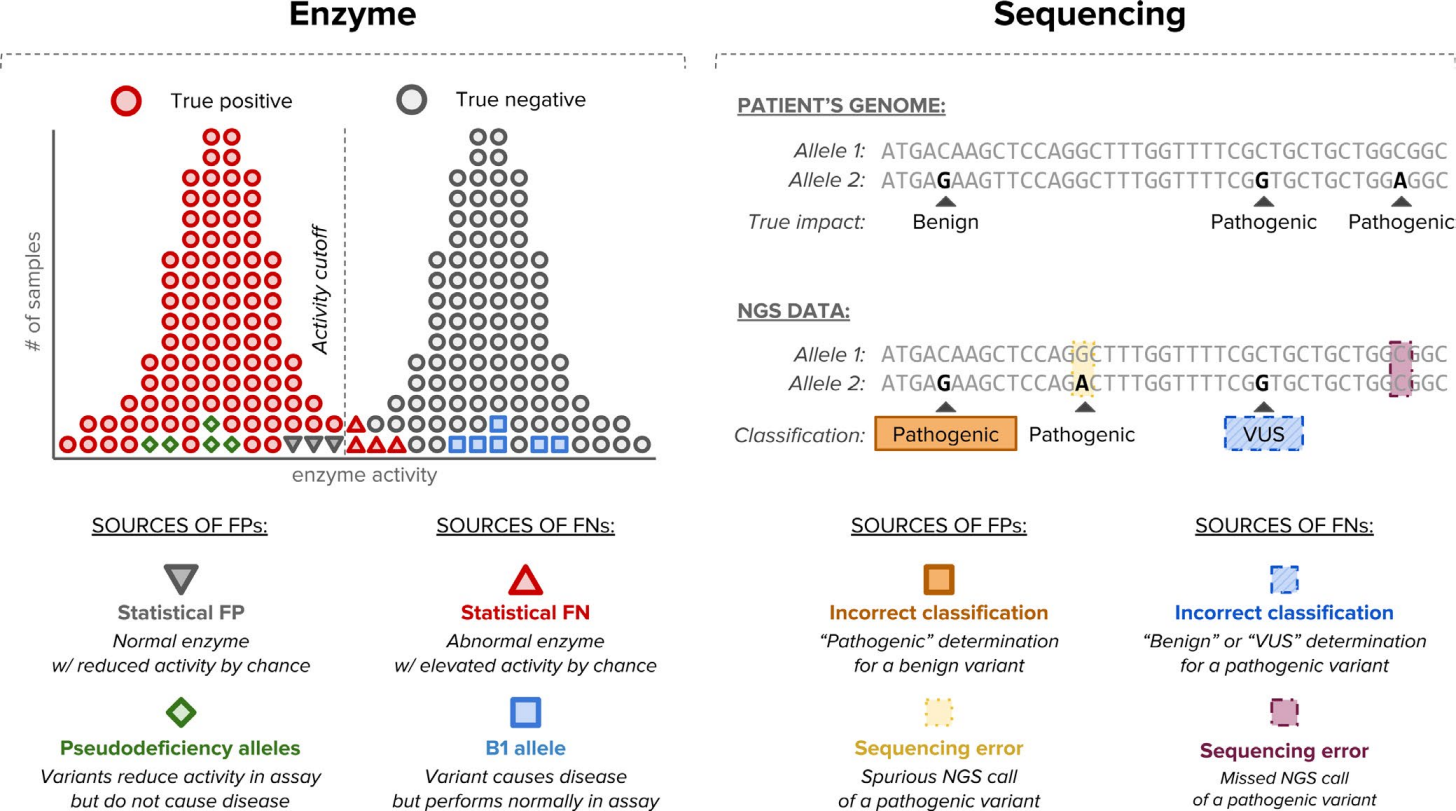
Sources of false positives and false negatives in Hex A enzyme‐based and *HEXA* NGS‐based carrier screening. Enzyme screening can yield false results due to statistical outliers (e.g., an impaired enzyme that randomly happens to yield activity above the assay threshold when tested) or well‐established variants (e.g., pseudodeficiency and B1 alleles) that are incompatible with the assay. NGS may produce false results due to incorrect variant classifications or analytical errors. Not depicted are additional potential sources of false screening results such as inconsistencies in enzyme level reference ranges across non‐AJ ethnicities and unidentified biologic factors. Enzyme activity levels near the cutoff are sometimes reported as “inconclusive” due to the statistical ambiguity. Abbreviations: FP, false positive; FN, false negative; NGS, next‐generation sequencing; VUS, variant of uncertain significance

For TSD, full‐exon NGS‐based carrier screening has several advantages compared to enzyme and targeted variant analysis, including the equitable analytical performance across ethnicities and capacity to correctly identify and classify B1 and pseudodeficiency alleles, which respectively lead to false negatives and false positives by enzyme analysis (Figure 1). Additionally, DNA‐based testing allows for more flexibility in sample type (e.g., the option of testing saliva). Lastly, initial testing by DNA eliminates the need for a secondary screening assay to resolve carrier status, a step which is necessary when enzyme results fall in the inconclusive range. However, NGS is a viable alternative to enzyme testing only if it achieves comparable or superior clinical performance in terms of sensitivity and specificity, which in turn affect the PPV. Because they could impact clinical sensitivity, specificity, and PPV, variants of uncertain significance (VUSs) have been cited as a shortcoming of NGS‐based TSD carrier screening (Hoffman et al., 2013). More specifically, because it is recommended that VUSs not be reported in the context of carrier screening, any pathogenic variant misclassified as VUS or benign would reduce the clinical sensitivity of NGS testing ((Edwards et al., 2015); Figure 1). Conversely, correct classification of existing *HEXA* VUSs as either pathogenic or benign can increase the clinical sensitivity and specificity of NGS‐based TSD screening.

Here we sought both to elucidate and improve the clinical test performance of NGS‐based TSD screening in a pan‐ethnic cohort by reclassifying *HEXA* VUSs. Reclassification efforts were approached in two ways: collection of biochemical phenotype data through Hex A enzyme analysis of patients harboring key VUSs, and reevaluation of previously classified *HEXA* VUSs using current ACMG‐AMP criteria. We describe the impact on test performance by estimating the clinical sensitivity, specificity, and PPV of *HEXA* sequencing for TSD carrier detection and comparing it to enzyme analysis.

## 2 METHODS

### 2.1 Ethical compliance

Eligible participants were consented by a JScreen/Emory genetic counselor under protocol number 00080928, approved by Emory University's Institutional Review Board.

### 2.2 Study population and design

Individuals in this study underwent *HEXA* sequencing as part of carrier screening ordered by their healthcare provider or through the JScreen program. Testing was completed at Myriad Women's Health (formerly Counsyl, South San Francisco). Informed consent was obtained by the individual's healthcare provider or by JScreen, and genetic counseling was made available to all individuals.

**Figure 2:**
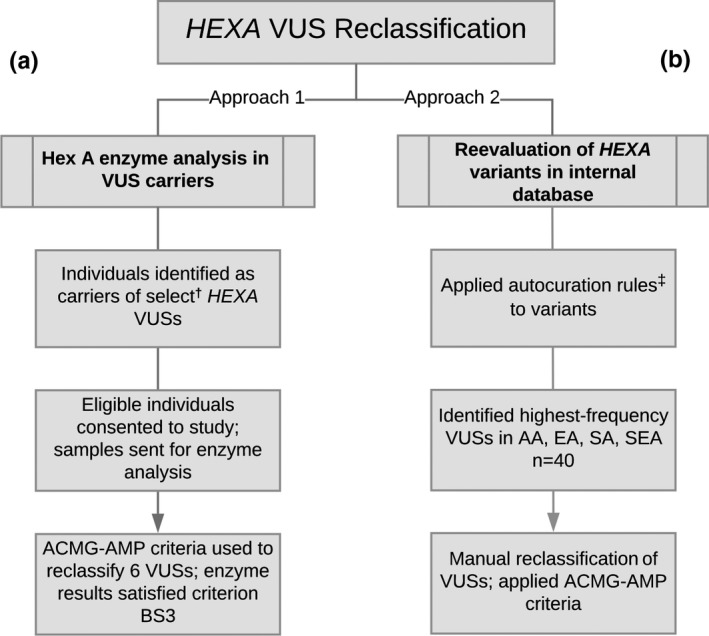
Two approaches to *HEXA* VUS reclassification. Study workflow demonstrating two approaches to variant reclassification. (a) Hex A enzyme analysis was performed in 29 individuals, each carrying one of six selected *HEXA* variants classified as a VUS at the time of testing. Enzyme results were used as functional evidence to satisfy the BS3 criterion in ACMG‐AMP guidelines during reclassification (Richards et al., 2015). (b) All *HEXA* variants in the Myriad Women's Health database were reevaluated. A standardized set of classification rules was applied to all variants, prompting some downgrades from VUS to likely benign. The remainder of VUSs were tabulated, and 40 VUSs were manually reevaluated in ethnicities with the highest VUS rates. Abbreviations: ACMG‐AMP, American College of Medical Genetics and Genomics‐Association for Molecular Pathology; AA, African American; EA, East Asian; SA, South Asian; SEA, Southeast Asian; VUS, variant of uncertain significance. †Variants shown in Table 2. ‡Classification framework described previously (Beauchamp et al., 2017)

Reclassification of *HEXA* variants of uncertain significance (VUSs) was performed via one of the two approaches (Figure 2). In the first approach, individuals carrying one of six *HEXA* VUSs were recruited and consented for subsequent Hex A enzyme analysis (Figure 2a). Each individual harbored a single *HEXA* variant—classified as a VUS at the time of testing—and had no other known or likely pathogenic *HEXA* variants or pseudodeficiency alleles. At the time of study design (2014–2015), six *HEXA* VUSs were observed at relatively high allele frequencies across ethnicities in Counsyl's database—c.8G>C (0.15%), c.253+5074C>T (0.44%), c.1074‐100T>C (0.22%), c.1074‐86G>A (0.02%), c.1397A>G (0.03%), c.1435G>A (0.24%)—and, thus, were selected as candidates for reclassification (Table S1). Individuals who reported a current pregnancy, history of bone‐marrow transplant, and/or treatment with antihypertensive medications were excluded to avoid potential interference with enzyme analysis (D’Souza et al., 2000; Lowden, Zuker, Wilensky, & Skomorowski, 1974). Those with self‐reported African American ancestry were excluded due to the lack of an ethnicity‐specific Hex A reference range for this population and the increased likelihood of receiving an inconclusive or false‐positive enzyme result (Mehta et al., 2016). Blood collection was coordinated, and samples were sent to the Mount Sinai Genetic Testing Laboratory (New York, NY) for Hex A enzyme analysis. Samples were transported for testing via priority overnight shipping to ensure expeditious processing and analysis. Upon completion of enzyme testing, a JScreen/Emory genetic counselor reviewed results with each participant via telephone or secure video conference. Each participant was awarded a gift card for participating in the study. Enzyme results were collated and incorporated into the variant reclassifications using ACMG‐AMP criteria (Richards et al., 2015).

The second approach (Figure 2b) to VUS reclassification entailed reevaluation of existing *HEXA* variants in the Myriad Women's Health database. After a set of interpretation rules were applied (Beauchamp et al., 2017), remaining variants classified as uncertain were tabulated. The 10 most common VUSs observed in ethnicities with the highest *HEXA* VUS rates (African American, East Asian, South Asian, Southeast Asian) were manually reevaluated by the Myriad Women's Health variant classification team in accordance with ACMG‐AMP criteria (Richards et al., 2015). *HEXA* variant allele frequencies were obtained from an anonymized cohort of *N* = 222,703 individuals undergoing routine carrier screening.

### 2.3 Hex A enzyme analysis

Hex A activity was assayed in both serum and white blood cells (WBCs). Testing was performed in accordance with the laboratory's protocol where Hex A% activity is measured by a previously described heat‐inactivation fluorometric method (Ben‐Yoseph, Reid, Shapiro, & Nadler, 1985). Reference ranges established by the testing laboratory were as follows (Hex A%): noncarrier WBC 55.0%–72.0%, noncarrier serum 58.0%–72.0%, carrier WBC <50%, carrier serum <54%. When Hex A values were discrepant between WBC and serum results, only the WBC‐derived values were reported, per the laboratory's protocol.

### 2.4 *HEXA* testing platform and variant classification

*HEXA* sequencing was performed as part of the Myriad Foresight Carrier Screen (previously Counsyl Family Prep Screen, South San Francisco). The laboratory was certified under the Clinical Laboratory Improvement Amendments (05D1102604), accredited by the College of American Pathologists Laboratory Accreditation Program (7519776), and received a permit from New York state (8535). Permitted samples included: whole blood, saliva, and extracted DNA. Testing was completed on an NGS platform employing hybrid capture followed by sequencing on Illumina instruments (Illumina, San Diego, CA) as previously described (Vysotskaia et al., 2017). Sequencing spanned exons 1‐14 (NM_000520.4), each padded by 20 flanking bases, and included targeted intronic positions where a ClinVar or HGMD entry existed at the time of assay design. Coverage of the 7.6 kb deletion prevalent in French Canadians was included (De Braekeleer, Hechtman, Andermann, & Kaplan, 1992). Variants were classified via a custom‐designed high‐throughput variant classification pipeline in accordance with ACMG‐AMP guidelines (Richards et al., 2015). The pipeline began with a rule‐based system to categorize variants based on their nature (e.g., molecular type such as SNP or indel, and consequence such as missense or nonsense), their minor allele frequency, and the availability of literature evidence. Variants without literature evidence were automatically classified (Beauchamp et al., 2017). Variants complex in nature and/or those with literature evidence required manual interpretation that included evaluation of primary lines of evidence—such as case reports, functional studies, and population frequency—identified through extensive literature search and review of public databases. Conservation data and predictions of both in silico structure and splicing were used as supporting evidence. Variant interpretations were subject to additional quality review and laboratory director approval before final classification. Per laboratory protocol for carrier screening, only pathogenic and likely pathogenic variants were reported. VUSs were not disclosed on the test report but were examined as part of this study.

### 2.5 Statistical analyses

#### 2.5.1 Power simulations for classification of VUSs based on Hex A% activity data

The number of negative enzyme results, *N_trials_*, required to reclassify a VUS as benign was estimated from a Monte Carlo analysis of a Gaussian model of Hex A% enzyme activities for carriers (mean 47%, standard deviation (SD) 4.8%) and noncarriers (mean 64%, SD 4.9%). Based on the historical internal rate at which VUSs are reclassified to pathogenic, a 0.31% prior probability of a classified VUS being clinically pathogenic was used. In each of 100,000 trials, *N_trials_* samples were drawn from the noncarrier distribution and the variant was considered benign if the posterior probability of a benign classification based on the *N_trials_* data points was greater than 99%. The fraction of trials that resulted in the correct classification was used to determine the probability of correct classification for each sample size (1 ≤ *N_trials_* ≤ 10). A similar process was used to estimate the minimum number of positive enzyme results required to classify a variant as pathogenic.

#### 2.5.2 Clinical sensitivity, specificity, and PPV estimation: NGS

The clinical sensitivity and specificity for NGS were estimated by assuming that false positives arise from incorrect pathogenic classifications and false negatives arise from incorrect non‐pathogenic classifications. The probabilities of these incorrect interpretations were estimated from historical internal reclassification‐rate data across 99 genes with the same criteria as *HEXA* (e.g., autosomal‐recessive conditions for which loss‐of‐function mutations are pathogenic); specifically, we considered variants for which the current classification differed from a past classification that was held for at least three months.

The probability of a true positive was calculated by weighting the overall probability of a pathogenic *HEXA* variant call (approximately twice the sum of observed *HEXA* allele frequencies for variants classified as pathogenic) by the probability that a general pathogenic variant is correctly classified (estimated from the historical internal rate of pathogenic classifications across the expanded carrier screen (ECS) that do not become reclassified as nonpathogenic).

The probability of a false negative was calculated by summing the following: (a) the overall probability of a VUS *HEXA* variant call weighted by the probability that a pathogenic variant is classified as a VUS (estimated from the historical internal rate of classifications changing from VUS to pathogenic), and (b) the overall probability of a rare (<1% allele frequency in all ethnicities) benign *HEXA* variant call weighted by the probability that a rare benign variant is classified as non‐benign (estimated from the historical internal rate of classifications for rare alleles changing from benign to pathogenic or from benign to VUS).

The clinical sensitivity was estimated by dividing the true‐positive probability by the sum of the true‐positive and false‐negative probabilities.

The probability of a true negative was calculated by summing the following: (a) the overall probability of a VUS *HEXA* variant call weighted by the probability that a VUS is benign (estimated from the historical rate of classifications that remain VUS or are downgraded from VUS to benign), (b) the overall probability of a rare benign *HEXA* variant call weighted by the probability that the benign classification is correct (estimated from the historical rate of classifications that remain benign), and (c) the probability that a patient does not screen positive for pathogenic, VUS, or rare benign variants (assuming that common benign variants are always correctly classified).

The probability of a false positive was calculated by weighting the overall probability of a pathogenic *HEXA* variant call by the probability that a pathogenic classification is incorrect (estimated from the historical rate of variant classifications changing from pathogenic to benign or VUS).

The clinical specificity was estimated by dividing the true‐negative probability by the sum of the true‐negative and false‐positive probabilities.

Carrier rates were estimated from allele frequencies obtained from a population with no clinical indication for testing other than routine carrier screening. The population labeled “non‐AJ” comprised individuals of any self‐reported ethnicity other than Ashkenazi Jewish, as well as those with unknown or unreported, mixed, and other ethnicities; this population reflects the non‐AJ individuals undergoing ECS at Myriad and not necessarily the non‐AJ U.S. population.

Analytical sensitivity and specificity were assumed to be perfect for the purposes of estimating clinical sensitivity and specificity. These assumptions are supported by analytical validation of NGS‐based ECS (Hogan et al., 2018).

PPV was estimated as the ratio of the probability of a true positive to the probability of a true positive or false positive.

Confidence intervals (95%) were estimated using the Clopper‐Pearson method (Clopper & Pearson, 1934).

#### 2.5.3 Clinical sensitivity, specificity, and PPV estimation: enzyme analysis

Sensitivity and specificity of enzyme testing—but not sequencing‐based assays—are affected by two types of systematic errors. First, B1 alleles have been reported as pathogenic (Kytzia & Sandhoff, 1985; Rozenberg et al., 2006; Tutor, 2004), but carriers of these variants do not screen positive on an enzyme‐based test (Figure 1). The probability of carrying the most common B1 allele—c.533G>A (p.Arg178His)—was counted toward reduced clinical sensitivity of enzyme‐based testing. Second, variants that have been reported as pseudodeficiency alleles are benign but show reduced enzyme activity due to lower activity on the test substrate compared to the biological substrate. The probability of carrying one of the two known pseudodeficiency alleles, c.739C>T (p.Arg247Trp) and c.745C>T (p.Arg249Trp) was counted toward reduced clinical specificity of enzyme‐based testing.

In addition to these systematic errors, statistical false positives and false negatives arise from decisions around the cutoff of enzyme activity levels. These types of errors were not included in this analysis of enzyme testing performance.

PPV was estimated as the ratio of the probability of a true positive (NGS carrier rate, minus the rate of B1 allele carriers) to the probability of a true positive or false positive (the carrier rate of pseudodeficiency alleles).

Confidence intervals (95%) were estimated using the Clopper–Pearson method (Clopper & Pearson, 1934). The rate of pathogenic variants observed in the sequencing assay was used as the prevalence in sensitivity and specificity estimations for enzyme testing.  For ratios of sensitivity, specificity, PPV, and NPV used to compare NGS and enzyme analysis, confidence intervals and *p*‐values were determined using a Z‐test on the log‐transformed risk ratios.

## 3 RESULTS

### 3.1 Reclassification of *HEXA* variants of uncertain significance

Multiple lines of evidence were evaluated to reclassify *HEXA* variants in accordance with ACMG‐AMP criteria. ACMG‐AMP criteria establish that in vitro functional studies like Hex A enzyme analysis can help to elucidate pathogenicity (criteria PS3 and BS3); therefore, Hex A% activity was assayed in 29 individuals heterozygous for one of six variants classified as VUS at the time of study design (2014–2015). Power analysis suggested that four to five independent negative enzyme results for a single VUS were required to achieve greater than 99% confidence in correctly reclassifying a VUS to benign (B) or likely benign (LB) (see Methods). Thus, five independent samples were evaluated for five of the VUSs and four participant samples for one VUS (Table 1). Twenty participants reported AJ ancestry, five reported Southeast Asian background, and ancestry information was unavailable for the remaining four patients. Hex A% activity was measured in serum and WBCs with the exception of three participants who reported oral contraceptive or hormone use that renders serum analysis inadequate; in these cases, analysis was performed on WBCs only. Hex A results revealed negative carrier status for all participants, satisfying the BS3 criterion. All six VUSs were reclassified to benign or likely benign based on the criteria outlined in Table 2. As of April 2018, only two of the six variants had ClinVar entries from submitters other than Counsyl: c.1435G>A (p.Ala479Thr) and c.1397A>G (p.Asn466Ser). EGL Genetics submitted a classification of benign (2016) for c.1435G>A (p.Ala479Thr), concordant with our reclassification. Both Illumina (2016) and EGL Genetics (2013) classified the variant, c.1397A>G (p.Asn466Ser), as a VUS (Table 2). Each laboratory provided their criteria for classification, although summary evidence was not supplied.

**Table 1:** Enzyme values in *HEXA* VUS heterozygotes

HGVS cDNA; Amino Acid	Participant ID	Ethnicity^a^	% Hex A activity (WBC)	% Hex A activity (serum)	Hex A result interpretation^b^
c.8G>C; p.Ser3Thr	1	AJ	69.0	73.3	Noncarrier
	2	unc	69.1	74.7	Noncarrier
	3	AJ	60.4	N/A^c^	Noncarrier
	4	AJ	62.9	58.9	Noncarrier
	5	AJ	56.8	62.9	Noncarrier
c.253+5074C>T	6	AJ	65.1	67.5	Noncarrier
	7	unc	59.5	59.3	Noncarrier
	8	AJ	63.1	63.0	Noncarrier
	9	AJ	58.9	73.4	Noncarrier
	10	AJ	58.6	58.6	Noncarrier
c.1074‐100T>C	11	AJ	68.1	71.5	Noncarrier
	12	AJ	56.6	69.1	Noncarrier
	13	AJ	55.1	67.9	Noncarrier
	14	AJ	66.4	69.6	Noncarrier
	15	AJ	68.1	67.8	Noncarrier
c.1074‐86G>A	16	unc	59.5	N/A^c^	Noncarrier
	17	unc	62.2	63.7	Noncarrier
	18	AJ	64.3	59.1	Noncarrier
	19	AJ	56.4	62.9	Noncarrier
c.1397A>G; p.Asn466Ser	20	AJ	56.6	60.1	Noncarrier
	21	AJ	58.5	59.0	Noncarrier
	22	AJ	70.7	58.8	Noncarrier
	23	AJ	57.2	N/A^c^	Noncarrier
	24	AJ	66.7	63.7	Noncarrier
c.1435G>A; p.Ala479Thr	25	SEA	55.8	60.1	Noncarrier
	26	SEA	66.9	65.1	Noncarrier
	27	SEA	64.8	N/A^c^	Noncarrier
	28	SEA	67.1	56.9	Noncarrier^d^
	29	SEA	58.6	65.6	Noncarrier

Note

RefSeq: NM_000520.4.

Abbreviations: AJ, Ashkenazi Jewish; N/A, not applicable; SEA, Southeast Asian; unc, uncertain.

a Self‐reported.

b Expected noncarrier range: 55.0–72.0 (WBC); 58.0–72.0 (serum). Inconclusive range: 50.0–54.9 (WBC); 54.0–57.9 (serum). % Hex A activity above 72.0 interpreted as non‐carrier.

c If WBC is in the noncarrier range and serum is discrepant, only the WBC value was reported and the patient was classified as a noncarrier.

d % Hex A within noncarrier range for WBC; inconclusive by serum. Participant reported oral contraceptive use at time of testing, a known contraindication for Hex A serum analysis.

**Table 2:** *HEXA* VUS Reclassifications through enzyme analysis

HGVS cDNA; amino acid	Molecular consequence	ACMG criteria met	Rule(s) used for classification	ACMG reclassification	ClinVar submission(s)^a^	ClinVar discrepancy^b^
c.8G>C; p.Ser3Thr	Missense	BS2, BS3^c^, BP4	Classification based on≥2 Strong (BS1–BS4)	Benign	1. Counsyl (8/31/17)	N/A
c.253+5074C>T	Intron	BS2, BS3^c^, BP4	Classification based on≥2 Strong (BS1–BS4)	Benign	1. Counsyl (8/31/17)	N/A
c.1074‐100T>C	Intron	BS3^c^, BP4, BP6	Classification based on 1 Strong (BS1–BS4) and 1 supporting (BP1– BP7)	Likely benign	1. Counsyl (8/31/17)	N/A
c.1074‐86G>A	Intron	BP4, BP6	Classification based on≥2 Supporting (BP1–BP7)	Likely benign	1. Counsyl (8/31/17)	N/A
c.1397A>G; p.Asn466Ser	Missense	BS3^c^, BP4	Classification based on 1 Strong (BS1–BS4) and 1 supporting (BP1– BP7)	Likely benign	1. Counsyl (8/31/17) 2. Illumina (6/14/16) 3. EGL Genetic Dx (9/18/13)	Likely benign VUS VUS
c.1435G>A; p.Ala479Thr	Missense	BS2, BS3^c^, BP6	Classification based on ≥ 2 Strong (BS1–BS4)	Benign	1. Counsyl (8/31/17) 2. EGL Genetic Dx (4/15/16)	N/A

Note

RefSeq: NM_000520.4.

Abbreviations: N/A, not applicable; VUS, variant of uncertain significance.

a All publically published ClinVar submissions (https://www.ncbi.nlm.nih.gov/clinvar/) as of 04/24/2018. Submission dates noted in parentheses.

b Classification submitted to ClinVar by corresponding laboratory. N/A denotes submissions with no discordance either because submitters agree on classification or only one submission exists for variant.

c BS3 functional studies criterion described as “well‐established in vitro or in vivo functional studies shows no damaging effect on protein function or splicing” applied to noncarrier enzyme results, Richards et al., *Genetics in Medicine*, 2015.

Through additional efforts aimed at reclassifying previously identified variants in Myriad's database, we were able to further reduce the *HEXA* VUS rate. Of 480 total variants processed with a rule‐based variant‐interpretation pipeline, 27 were reclassified to likely benign due to their large distance from the nearest exon. The remaining VUSs were grouped and sorted by their ethnicity‐specific frequency. The highest residual VUS rates were observed in four ethnicities (African American, East Asian, South Asian, Southeast Asian), and the 10 most frequent VUSs for each ethnicity were selected for manual reevaluation. Of those 40 reevaluated VUSs, two had sufficient evidence to be reclassified as benign or likely benign (Table S5).

### 3.2 Clinical test performance of sequencing‐based *HEXA* carrier screening is equivalent or superior to enzyme‐based screening in an Ashkenazi Jewish and pan‐ethnic population

**Figure 3:**
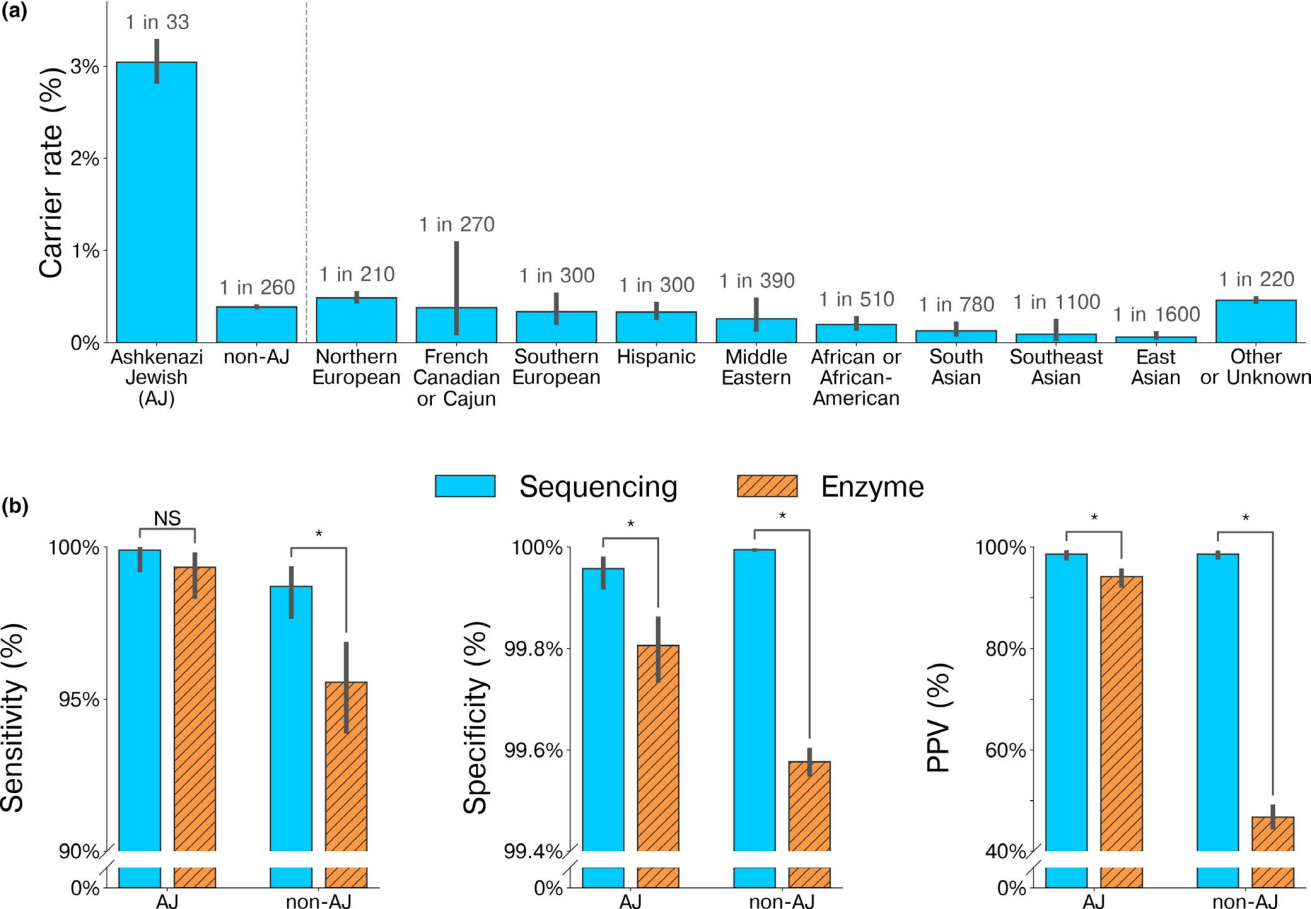
Clinical test performance of sequencing‐based *HEXA* carrier screening matches or exceeds that of enzyme‐based Hex A screening. (a) Population‐specific *HEXA* carrier rate estimated from NGS data. (b) The clinical sensitivity, specificity and positive predictive values of sequencing‐ and enzyme‐based *HEXA* carrier screening. The sensitivity and specificity of sequencing‐based carrier screening is reduced by potentially incorrect variant classifications, while enzyme‐based carrier screening has false negatives due to the B1 allele and false positives due to pseudodeficiency alleles (see Methods). Abbreviations: NS, not significant; PPV, positive predictive value. Error bars indicate 95% CI. Asterisk (*) indicates statistical significance (*p* < 0.05, see Methods)

Carrier frequencies observed in this study were 1/33 (95% CI, 1/30‐1/36) in the AJ population and 1/260 (1/240‐1/280) in the non‐AJ population, both consistent with published frequencies (Gross et al., 2008; Kaback et al., 1993) (Figure 3a, Table S2). Although the French Canadian or Cajun carrier frequency was found to be lower in this study (1/270) compared with previously reported frequencies, there is overlap between the 95% CI estimates of our study (1/91‐1/1300, *N* = 795) and the reported rate in a New England‐based French Canadian population (1/55‐1/107, *N* = 2,783) (Martin, Mark, Triggs‐Raine, & Natowicz, 2007) (Figure 3a). The difference (*p* = 0.022, Fisher's exact test) between the carrier rates observed here and those previously published may be explained by differences in population ascertainment as our cohort includes self‐ or physician‐reported ethnicities.

Using allele frequencies, variant classifications, and the historical rates of change in variant classifications, we estimated the clinical sensitivity, specificity, and PPV achievable with NGS‐based and enzyme‐based TSD screening assays (Figure 3b; see Methods). For each assay, we estimated test‐performance metrics for two cohorts: an AJ population (*N* = 19,637) and a pan‐ethnic population excluding AJs (*N* = 203,066). The latter is comprised of patients from nine self‐reported ethnicities: Northern European, Southern European, Hispanic, Middle Eastern, French Canadian or Cajun, African American, South Asian, Southeast Asian, and East Asian, as well as those with mixed, other, or no reported ethnicity (single‐ethnicity performance metrics in Table S4).

Clinical sensitivity for carriers was estimated to be 99.90% by NGS and 99.33% by enzyme testing in our AJ cohort, though the increased sensitivity of NGS was not statistically significant. However, in a pan‐ethnic population, estimated clinical sensitivity by NGS (98.70%; 95% CI, 97.64%‐99.37%) was significantly increased (*p* < 0.05, see Methods) compared to enzyme analysis (95.55%; 95% CI, 93.87%‐96.88%). In each population separately, the clinical specificity was estimated to be >99.9% by NGS‐based testing and >99.2% by enzyme‐based testing (Table S4; Figure 3b shows aggregated values). Compared to NGS, the estimated PPV for enzyme analysis was significantly lower in both AJ‐only and non‐AJ cohorts (*p* < 0.05, see Methods): among non‐AJs, estimated PPV was less than 50% for enzyme analysis and more than 98% for sequencing.

## 4 DISCUSSION

Enzyme testing for TSD carriers is a routine and standardized screening assay, yet DNA‐based testing can range from targeted variant analyses that identify a handful of ethnic‐specific variants to NGS strategies that can discover many thousands of variants. Although targeted DNA approaches are clinically inferior to enzyme testing in pan‐ethnic screening (Kaback et al., 1993; Park et al., 2010), the clinical accuracy of a strategy that pairs full‐exon NGS with guideline‐based interpretation of novel variants had not been previously explored. Here we reclassified VUSs in *HEXA* using a broad set of molecular and statistical evidence, and we revealed that the resultant estimated clinical sensitivity, specificity, and PPV of full‐exon NGS testing are equivalent or superior to those of enzyme analysis for both an AJ‐only and non‐AJ cohort.

Multiple different optimizations can increase the clinical sensitivity of an assay. For instance, improving analytical sensitivity—that is, the ability of the test to discover variants in patients’ genomes—can consequently increase clinical sensitivity by revealing the presence of variants that may be pathogenic (e.g., novel termination codons). However, Hogan et al. (2018) previously demonstrated that analytical sensitivity of NGS‐based carrier screening is>99.9% for single‐nucleotide variants and short insertions and deletions. Therefore, in order to maximize clinical sensitivity for TSD carriers, we sought not to increase the already‐proficient discovery of variants in *HEXA* but rather to decrease uncertainty about variant classifications. Principled reclassification of VUSs to benign or pathogenic improves clinical performance because VUSs are not reported to patients undergoing carrier screening (Edwards et al., 2015). Of the high‐frequency VUSs considered in this study, we reclassified all to either benign or likely benign, which ultimately increases clinical sensitivity for TSD carriers by lessening the chance of an unreported VUS being truly pathogenic (i.e., reducing the false‐negative rate).

The presence of B1 and pseudodeficiency alleles and the lack of established enzyme reference ranges across ethnicities impair the clinical test performance of enzyme analysis. However, neither limitation affects NGS‐based TSD screening. Because they are single‐base substitutions in the DNA that have no impact on the sequencing reaction itself, B1 and pseudodeficiency alleles yield neither false negatives nor false positives, respectively, in screening via NGS. The probability of carrying a B1 or pseudodeficiency allele was found to vary across ethnicities, consistent with previously reported gnomAD allele frequencies (Table S3). We used the ethnicity‐specific frequency data for these alleles from our NGS dataset to estimate the performance of enzyme testing in a pan‐ethnic clinical setting: importantly, sensitivities and specificities were lower in both the AJ and non‐AJ cohorts for enzyme‐based screening when compared to NGS‐based screening. Our estimates of enzyme test performance may nevertheless be optimistic because we did not attempt to model ethnicity‐specific reference ranges: the standard Hex A% reference range was originally established based only on the AJ population (Mehta et al., 2016; Strom et al., 2013), and ethnicity‐specific differences may cause false‐positive and inconclusive enzyme results (Mehta et al., 2016). Taken together, the lack of impact of B1 and pseudodeficiency alleles on NGS clinical test performance and the ability to produce results independent of enzyme reference ranges further underscore the technical and clinical advantages of applying NGS testing pan‐ethnically. These advantages manifest in the significantly elevated PPV of the NGS‐based assay in the non‐AJ group, which is more than double that of enzyme analysis (98.64% NGS; 46.74% enzyme analysis).

Over the past 10 years, professional medical societies, including ACOG and ACMG, have issued several carrier screening guidelines or position statements commenting on TSD screening (Table S6). Collectively, these publications support enzyme analysis as the preferred method for TSD carrier detection across populations. However, upon review of the literature, including references cited in the aforementioned guidelines and statements, no studies were identified that either evaluated or supported the claim that enzyme analysis provides superior carrier detection across ethnicities. While previously published studies determined that enzyme analysis has 98% sensitivity in the AJ population (Kaback et al., 1993), this sensitivity level has not been established in other populations. Furthermore, when making test‐performance comparisons between DNA‐based testing and enzyme analysis, it is critical to consider the methodology employed by the DNA‐based screen, as it is already well‐established that targeted variant analysis for TSD does not perform adequately in a pan‐ethnic population (Kaback et al., 1993; Park et al., 2010). Guidelines published as recently as 2017, including ACOG Committee Opinions 690 and 691, reference the limitations of targeted DNA variant testing but do not comment on the performance of NGS‐based TSD screening. Our demonstration that NGS‐based testing has equivalent or superior estimated clinical sensitivity, specificity, and PPV relative to enzyme testing suggests that the guidelines supporting enzyme testing should be revisited to account for the different modes of DNA‐based testing and their respective efficacies.

As this study performed estimation of clinical performance based on variant reclassification data and retrospectively gathered allele frequencies, it is not free of limitations. Specifically, real performance metrics may differ from our estimated values if variant frequencies and reclassification analyses are incorrect. We attempted to limit bias in variant frequencies by restricting the analysis to those without family history of disease (i.e., only patients undergoing routine testing were analyzed); further, we measured frequencies in a large cohort of 222,703 patients. However, future studies involving larger cohorts of ethnicities that were relatively sparsely sampled in our dataset (e.g., French Canadians) could improve the test‐performance estimates. Error in estimating *HEXA* variant‐reclassification rates could also impact our estimated NGS test performance, though we attempted to minimize this effect by calculating average rates over 99 genes that resemble *HEXA*; this average should be free from small sample size artifacts but could nevertheless be offset from the real values for *HEXA*. Lastly, the reclassification of one variant, c.1397A>G, required enzyme results for reclassification to likely benign. Relying upon enzyme testing for this reclassification may cause concern given the known limitations of enzyme testing, such as the B1 allele yielding a false‐negative result. However, in the case of c.1397A>G, the high frequency of this variant in the AJ population (0.168%) strongly argues against it being pathogenic because ~10% of affected patients would be expected to harbor the variant, and it has not been observed at that rate among the many hundreds of TSD‐affected patients characterized in the literature (see Data S1). Further, if this allele were actually pathogenic but yielded a false‐negative enzyme result (like the B1 allele) that caused a reclassification error, our estimates of clinical sensitivity, specificity, and PPV would be equivalently overinflated for both NGS and enzyme testing. Notably, enzyme results were not strictly required for reclassification of other *HEXA* variants in this study because sufficient orthogonal supporting evidence was available.

The retrospective nature of our study could be viewed as a limitation but was also a practical necessity. Evaluating clinical test performance prospectively would be prohibitive given the low worldwide TSD prevalence, which is largely due to the success of population‐based screening programs (Kaback et al., 1993). Calculating clinical sensitivity in obligate carriers or affected individuals in a side‐by‐side comparison of NGS‐ and enzyme‐based testing would be impeded by recruitment difficulties and take many years to achieve statistical significance. Furthermore, the available sample population would not be sufficient to make statistically significant claims about assay performance in both AJ‐only and pan‐ethnic cohorts.

## 5 CONCLUSION

As the established goal of carrier screening is to provide couples with results that inform reproductive decision‐making, the importance of offering a TSD screen that performs accurately and equitably in patients of any ethnicity is integral. Overall, the data we present provide evidence and support for NGS‐based screening as the optimal method to identify TSD carriers, irrespective of ethnicity.

## CONFLICT OF INTEREST

A.C.C., K.E.K., L.K., K.M, and D.M. are current or former employees of Myriad Women's Health, a laboratory that performs expanded carrier screening. N.M and I.S.H. are former Counsyl employees. All other authors declare no conflicts of interest.
