# Supplementary material for: Screening for Tay‐Sachs disease carriers by full‐exon sequencing with novel variant interpretation outperforms enzyme testing in a pan‐ethnic cohort
Source: Mol Genet Genomic Med. 2019 Jul 10;7(8):e836. doi: 10.1002/mgg3.836 (PMC6687860; doi:10.1002/mgg3.836)
Supplement: Supplementary file 1 [file MGG3-7-e836-s001.pdf]

Additional argument supporting non-pathogenic classification of c.1397A>G

As an additional evaluation of whether the c.1397A>G variant is pathogenic, we pursued the following approach: assume it is pathogenic and then test whether corollaries of that assumption are inconsistent with other data.

We observed that the c.1397A>G variant has an allele frequency of 0.168% in the AJ population. Further, we observed that 598 of 19637 AJ patients are carriers of other pathogenic variants. Because each carrier has two alleles, the frequency of already-established pathogenic alleles is 598 / (2 * 19637) = 1.522%. If c.1397A>G were also a pathogenic allele, then it would be expected to account for (0.168% / (1.522% + 0.168%)) = 9.9% of carriers, and the variant's expected frequency in affected patients would be comparable. In the process of performing variant classification on *HEXA*, we have identified nearly 400 TSD case reports in the literature. These case reports are accompanied by the putative causal variants. If c.1397A>G were pathogenic, then we would expect ~40 of these affected patients (9.9% * 400) to have the c.1397A>G variant reported as a causal variant, yet the variant was reported as such in none of the cases. Therefore, because a simple inference of our assumption is strongly inconsistent with observed data, we reject the original assumption that the c.1397A>G is pathogenic.
